# The Phytochemical Agathisflavone Modulates miR146a and miR155 in Activated Microglia Involving STAT3 Signaling

**DOI:** 10.3390/ijms25052547

**Published:** 2024-02-22

**Authors:** Balbino Lino dos Santos, Cleonice Creusa dos Santos, Karina Costa da Silva, Carolina Kymie Vasques Nonaka, Bruno Solano de Freitas Souza, Jorge Mauricio David, Juciele Valéria Ribeiro de Oliveira, Maria de Fátima Dias Costa, Arthur Morgan Butt, Victor Diogenes Amaral da Silva, Silvia Lima Costa

**Affiliations:** 1 Laboratory of Neurochemistry and Cellular Biology, Institute of Health Sciences, Federal University of Bahia, Av. Reitor Miguel Calmon S/N, Salvador 40231-300, BA, Brazil; cleonicemev@gmail.com (C.C.d.S.); karinacostads@gmail.com (K.C.d.S.); juciele.valeria@ufba.br (J.V.R.d.O.); fatima@ufba.br (M.d.F.D.C.); vdsilva@ufba.br (V.D.A.d.S.); 2 College of Nursing, Federal University of Vale do São Francisco, Av. José de Sá Maniçoba, S/N, Petrolina 56304-917, PE, Brazil; 3 Center of Biotechnology and Cell Therapy, São Rafael Hospital, D’Or Institute for Research and Teaching (IDOR), Salvador 41253-190, BA, Brazil; carolina.nonaka@hsr.com.br (C.K.V.N.); bruno.solano@idor.org (B.S.d.F.S.); 4 Instituto Gonçalo Moniz, Fundação Oswaldo Cruz (FIOCRUZ), Salvador 40296-710, BA, Brazil; 5 Department of General and Inorganic Chemistry, Institute of Chemistry, Federal University of Bahia, Salvador 40231-300, BA, Brazil; jmdavid@ufba.br; 6 National Institute of Translational Neuroscience (INNT), Rio de Janeiro 21941-971, RJ, Brazil; 7 School of Pharmacy and Biomedical Sciences, University of Portsmouth, Portsmouth PO1 2DT, UK; arthur.butt@port.ac.uk; 8 Instituto de Ciências da Saúde, Av. Reitor Miguel Calmon S/N Vale do Canela, Salvador 40110-902, BA, Brazil

**Keywords:** agathisflavone, human microglia, anti-inflammatory, miRNAs, STAT3

## Abstract

MicroRNAs (miRs) act as important post-transcriptional regulators of gene expression in glial cells and have been shown to be involved in the pathogenesis of neurodegenerative diseases, including Alzheimer’s disease (AD). Here, we investigated the effects of agathisflavone, a biflavonoid purified from the leaves of *Cenostigma pyramidale* (Tul.), on modulating the expression of miRs and inflammatory mediators in activated microglia. C20 human microglia were exposed to oligomers of the β-amyloid peptide (Aβ, 500 nM) for 4 h or to lipopolysaccharide (LPS, 1 µg/mL) for 24 h and then treated or not with agathisflavone (1 µM) for 24 h. We observed that β-amyloid and LPS activated microglia to an inflammatory state, with increased expression of miR-146a, miR-155, IL1-β, IL-6, and NOS2. Treatment with agathisflavone resulted in a significant reduction in miR146a and miR-155 induced by LPS or Aβ, as well as inflammatory cytokines IL1-β, IL-6, and NOS2. In cells stimulated with Aβ, there was an increase in p-STAT3 expression that was reduced by agathisflavone treatment. These data identify a role for miRs in the anti-inflammatory effect of agathisflavone on microglia in models of neuroinflammation and AD.

## 1. Introduction

Microglial activation to a proinflammatory profile plays a critical role in neurodegeneration, as occurs in Alzheimer’s disease (AD). Proinflammatory microglia express cytokines, chemokines, and other mediators involved in the regulation of neuroinflammation, such as microRNAs [1,2]. MicroRNAs (miRNAs) are endogenous, short, noncoding RNA molecules that act as important post-transcriptional regulators of gene expression by base-pairing with target mRNA [3,4]. Multiple studies have demonstrated miRNAs as important regulators and risk factors for the development of neurodegenerative diseases, including Alzheimer’s and Parkinson’s diseases [5,6,7]. Furthermore, miRNAs have been shown to regulate the JAK2/STAT-3 signaling pathway and consequent modulation of inflammatory responses induced in microglia [8,9]. The miRNA miR-155, for example, has been associated with the suppression of inflammatory cytokines via reduction of p-STAT-3 [10]. On the other hand, in a study by Zhao et al. [11], the role of microRNA-146a in modulating anxiety-like behavioral activity in mice and oxidative stress and neuroinflammation was demonstrated.

Flavonoids are a group of polyphenolic compounds from different plant species with varied biological effects, such as anti-inflammatory, antioxidant, antitumor, antimicrobial, and antiviral actions [12,13,14,15,16,17,18,19]. The neuroprotective effects of flavonoids may be related to their action on the expression of miRNAs and their proinflammatory properties in microglial cells [3,20,21]. Our previous studies have demonstrated that the biflavonoid agathisflavone, purified from the leaves of *Cenostigma pyramidale* (Tul.) (syn. *Caesalpinia pyramidalis* and *Poincianella pyramidalis*), is neuroprotective [22] and antineuroinflammatory, which was mainly associated with modulation of the microglial inflammatory profile [23,24,25]. In this context, flavonoids have been shown to downregulate microglial proinflammatory state in both the lipopolysaccharide (LPS) and Aβ models of neuroinflammation and AD [25,26]. However, the effect of flavonoids on regulating microRNAs and microglial activation is poorly understood. Here, we provide evidence that the flavonoid agathisflavone modulates the neuroinflammatory response of human microglia through the regulation of miR-146a and miR-155.

## 2. Results

### 2.1. Agathisflavone Modulates the Microglial Activation Profile Induced by β-Amyloid Oligomers and LPS

**Figure 1:**
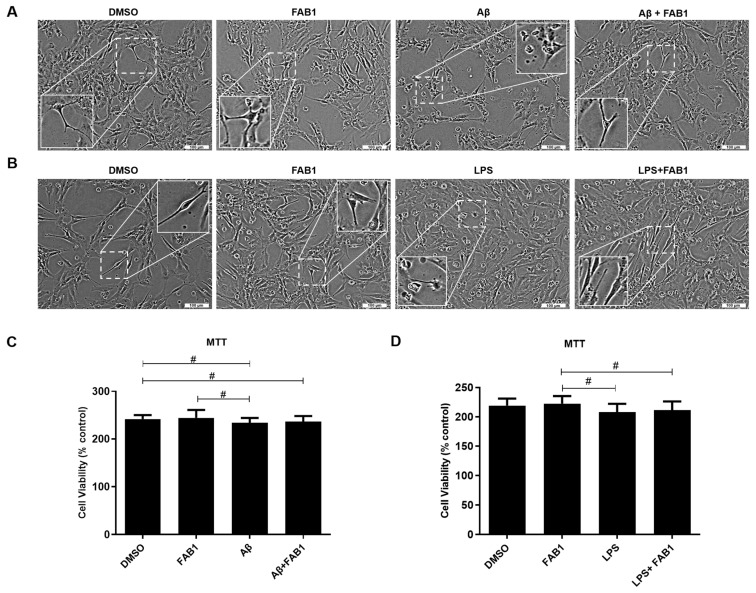
Effects of agathisflavone on human microglia treated with β-amyloid or LPS. C20 human microglia cells were exposed to β-amyloid (500 nM) for 4 h or to LPS (1 µg/mL) for 24 h and/or treated with (1 µM) of agathisflavone (FAB1) for 24 h. Control cultures were treated only with DMSO vehicle (0.001%). (**A**,**B**) Phase contrast photomicrographs of microglial cells in the different treatment groups; scale bar = 100 µm. (**C**,**D**) MTT analysis of microglial cell viability in the different treatment groups; results expressed relative to controls (100%) and tested for significance by one-way ANOVA; # *p* < 0.05, and bars indicate comparisons between the different conditions. Aβ: β-amyloid; FAB1: agathisflavone; LPS: lipopolysaccharide.

Here, we evaluated the effect of agathisflavone (1 µM) on cell morphology and viability, using phase contrast microscopy and the MTT assay (Figure 1). The concentration and exposure time adopted was based on our previous studies that demonstrated neuroprotective and immunomodulatory effects of the flavonoid in vitro [25]. In the control cultures (0.001% DMSO only) and in those treated with the flavonoid alone, the microglia had a predominantly multipolar and branched morphology, with thin and long processes extending from the cell bodies (Figure 1A,B). In contrast, microglial treated with LPS or Aβ exhibited a more amoeboid morphology, with larger cell bodies and retraction of cytoplasmic processes, which were short and thick. Furthermore, we observed many rounded cells and cell debris typical of apoptosis (Figure 1A,B). These alterations in microglial morphology induced by LPS- or Aβ- were barely evident following cotreatment with agathisflavone, with the presence of more branched microglia observed in controls (Figure 1A,B). Results from the MTT assay demonstrated a significant decrease in microglial cell viability after LPS treatment, and although there was also decreased viability in Aβ, this was not statistically significant. On the other hand, after treatment with the flavonoid, we noticed an increase in viability (Figure 1C,D) and we observed, in phase contrast microscopy, a prevalence of more branched and less reactive cells (Figure 1A,B).

### 2.2. Agathisflavone Regulates miRNA Expression and Reduces Microglial Activation

**Figure 2:**
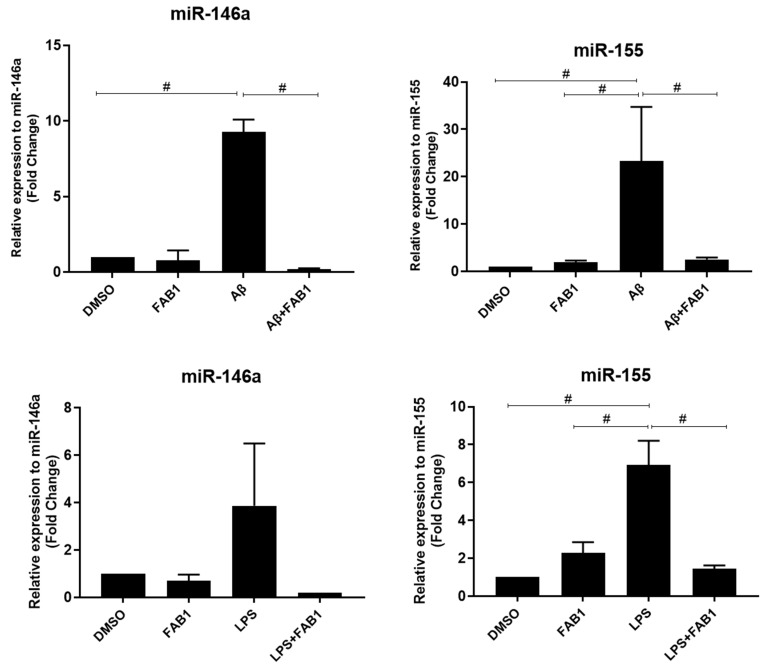
Effects of agathisflavone on microRNAs (miRNAs) of human microglia treated with β-amyloid or LPS. C20 lineage human microglia cultures were exposed to β-amyloid (500 nM) for 4 h or to LPS (1 µg/mL) for 24 h and/or treated with 1 µM of agathisflavone (FAB1) for 24 h. Control cultures were treated only with DMSO (0.001%). RT-qPCR for miRNA was performed to determine relative expression levels of miRNA-146a and miRNA-155. Data presented as mean change ± SEM times from controls. They were tested for significance by one-way ANOVA; # *p* < 0.05 (n = 3), and bars indicate comparisons between the different conditions. Aβ: β-amyloid; FAB1: agathisflavone; LPS: lipopolysaccharide.

Next, we evaluated cultures of agathisflavone on the expression of miR146a and miR155 in microglia activated by Aβ or LPS. C20 human microglia cells showed a significant increase in the expression of miR-146a and miR-155 following treatment with Aβ or LPS, and this was completely blocked by cotreatment with agathisflavone (Figure 2).

### 2.3. Agathisflavone Regulates the Expression of Inflammatory Cytokines in Human Microglia Cultures Subjected to Aβ Damage

**Figure 3:**
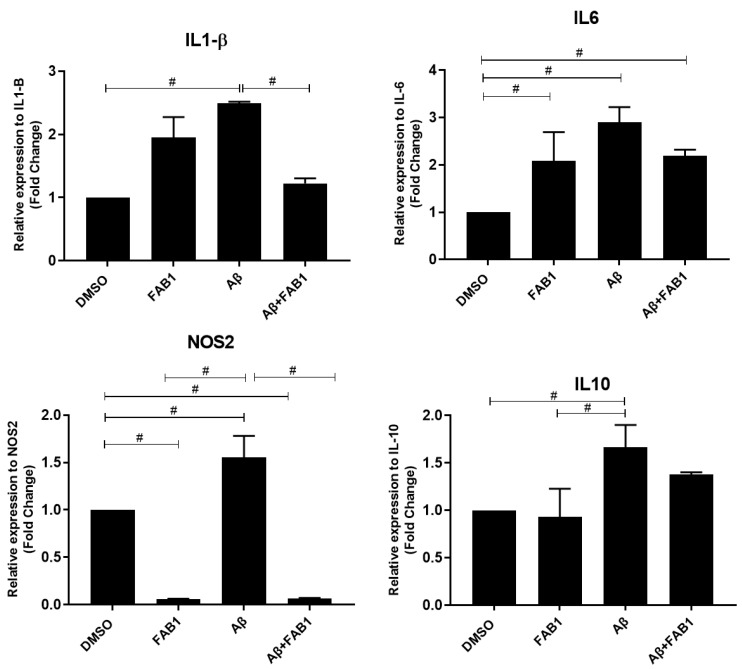
Effects of agathisflavone on inflammatory genes in β-amyloid-treated microglia. C20 lineage human microglia cultures were exposed to β-amyloid (500 nM) for 4 h and/or treated with 1 µM agathisflavone (FAB1) for 24 h and RT-qPCR was performed on microglial cells to determine relative expression levels of mRNA for the inflammatory mediators IL1β, IL6, NOS2, and anti-inflammatory cytokine IL-10. Data presented as mean ± SEM fold change relative to controls. They were tested for significance by one-way ANOVA; # *p* < 0.05 (n = 3), and bars indicate comparisons between the different conditions. Aβ: β-amyloid; FAB1: agathisflavone.

RT-qPCR evaluation of the expression of the inflammatory cytokines IL-1β, IL-6, and NOS2 and the anti-inflammatory cytokine IL-10 demonstrated that these were all significantly altered following activation of microglia with Aβ, compared to control cultures, and the changes in proinflammatory cytokines were significantly reduced by agathisflavone (Figure 3). The greatest effect was on NOS2, which was completely inhibited by agathisflavone. In comparison, agathisflavone had no significant effect on the anti-inflammatory cytokine IL-10, which was increased by Aβ.

### 2.4. Agathisflavone Modulates STAT-3/pSTAT-3 Expression in Microglia Cells Induced by Aβ

**Figure 4:**
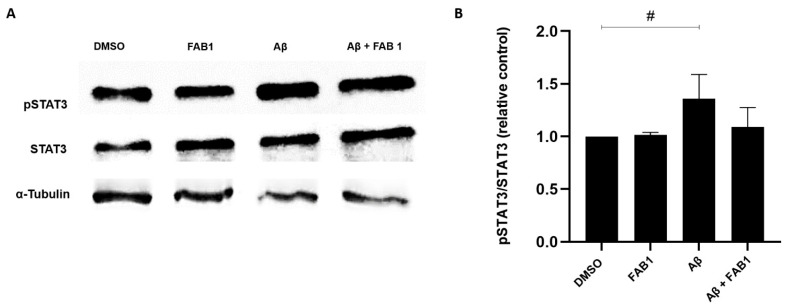
Effects of agathisflavone on the pSTAT-3/STAT-3 signaling pathway in human microglia treated with β-amyloid. Cultures of microglia cells were exposed to β-amyloid (500 nM) for 4 h and/or treated with 1 µM of agathisflavone (FAB1) for 24 h. Samples of 15 μg of total protein were run electrophoretically and Western blot analysis for pSTAT-3/STAT-3 was performed on a 10% polyacrylamide gel. (**A**) Representative Western blots showing that FAB treatment downregulated pSTAT3 expression in microglial cells. (**B**) Quantification of pSTAT3 presented as fold changes. Data presented as mean change ± SEM times of controls. They were tested for significance by one-way ANOVA; # *p* < 0.05 (n = 3). Aβ: β-amyloid; FAB1: agathisflavone.

Finally, we assessed whether the flavonoid agathisflavone modulated the expression of STAT3 and p-STAT3, because of the importance of this signaling protein in the regulation of inflammatory processes and in the expression of cytokines. For this, primary cultures of rat microglia were treated with Aβ for a period of 4 h, which significantly increased the p-STAT3/STAT3 ratio, compared to cells treated only with the vehicle DMSO, and this was reduced by treatment with agathisflavone (1 µM) (Figure 4)**.**

## 3. Discussion

Microglia play a central role in the immune defense of the central nervous system (CNS). In their resting state, microglia have a well-branched morphology and interact with neurons, other glial cells, and the surrounding microenvironment, performing immunological surveillance and contributing to the maintenance of CNS homeostasis [27]. When there is any insult to the CNS, such as occurs in neurodegenerative diseases, microglia become activated, which involves a transformation of their morphology, proliferative state, migration, and the release inflammatory mediators, including cytokines and chemokines that are harmful to the CNS [13]. In the present work, we used a model of neuroinflammation induced by Aβs and LPS in C20 human microglia cultures and evaluated the potential of the biflavonoid agathisflavone to modulate the microglial inflammatory response. Our results show that Aβ and LPS induced microglial activation, as characterized by morphological changes and cytokine profile, and this was related to an increase in miRNA-146a and miRNA-155. Notably, treatment with the flavonoid agathisflavone decreased miRNA 146a and 155 and inhibited microglial activation induced by Aβ and LPS. The results support a role for miRNAs in mediating microglia activation and indicate that they are an important target in the antineuroinflammatory effects of agathisflavone in microglia.

The flavonoid agathisflavone significantly inhibited microglial activation, maintaining their ramified morphology, and decreased proinflammatory state, with a marked inhibition of NOS2. In a previous study carried out in a neuroinflammation model using primary culture of rat microglia, we demonstrated that the flavonoid agathisflavone reduced the expression of inflammatory mediators and negatively regulated the expression of the NLRP3 inflammasome complex in rat microglia cultures submitted to LPS damage [25]. Furthermore, we also observed that microglia cultures submitted to LPS damage and treated with agathisflavone showed a prevalence of less reactive microglia, presenting a more branched morphology [25]. A similar effect on the microglial profile was also observed in cocultures of neurons and glial cells submitted to inflammatory damage with LPS, IL1β, or Aβ and treated with agathisflavone monomer apigenin, in which the neuroprotective and immunomodulatory effects were evidenced [26].

In normal CNS, microglia are in a nonactivated state, and when activated they undergo morphological changes, proliferate, migrate by chemotaxis, and are induced to produce cytokines and chemokines involved in inflammatory and immunomodulatory responses. The stimuli and mechanisms involved in this process of microglial activation are highly complex and not fully understood. Currently, there is a growing number of studies on the involvement of miRNAs and their relationship with the microglial response. Alterations in the regulation of miRNA molecules in activated microglia cells, for example, have contributed to the development and progression of neurodegenerative diseases and brain injuries [28,29].

MicroRNAs emerge as new targets in investigations of the regulatory mechanisms of numerous cellular processes, as they act as regulators that repress the expression of target genes [1,27]. Studies have shown that many of these miRNAs are associated with the regulation of growth, development, and regulation of cellular processes such as differentiation, metastasis, and proliferation [30,31]. Furthermore, it has also been shown that the proinflammatory microglial phenotype is characterized by upregulation of miR-155 and miR-146a, related to the release of inflammatory cytokines, including IL-1β and NO [3], which we show here are increased in microglia by LPS and Aβ, and inhibited by agathisflavone.

Notably, activation of human C20 microglial cells with LPS or Aβ increased miR-146a expression, with increases in IL-6, IL-1β, and NOS2, implicating miRNA-146a in regulating the expression of inflammation mediators [32]. This is supported by the effects of agathisflavone, which decreased the rise in miRNA-146a in LPS- and Aβ-treated microglia, and at the same time reduced microglial activation, completely inhibiting the increase in NOS2 expression. Our results are consistent with studies indicating that miR-146a modulates the innate immune response and neuroinflammation, downregulating the production of proinflammatory cytokines and their mediators in both microglial and astrocytic cells, involving NF-κB [1,32,33,34,35]. Increased miR146a expression in BV-2 microglial cells stimulated with LPS has previously been suggested in the negative regulation of microglial activation [36]. Cytokines such as IL-1β have also been shown to induce miR-146a expression, leading to downregulation of human-astrocyte-mediated neuroinflammatory response [2]. An upregulation of miRNAs 146a, 155, and 132 has been shown in human THP-1 macrophages stimulated with LPS, with the IRAK1 and TRAF6 genes being the targets of post-translational repression by miR-146 and involving TLR2 and TLR4 receptors in the positive expression of miR-146a [32,35].

In addition, we also observed a marked increase in miR-155 in microglia treated with Aβ or LPS, and this increase was significantly reduced (*p* < 0.05) by agathisflavone. The results further indicate a direct relationship between upregulation of miR-155 and microglial activation in response to neuroinflammatory mediators, and in the anti-inflammatory effects of this flavonoid on microglia. Our results are consistent with studies in mouse models of AD induced by Aβ, showing increased expression of miR-155 and microglial activation, together with an increase in the secretion of inflammatory cytokines [27,37]. Similarly, in a study with BV2 microglial cells, it was shown that treatment with LPS positively regulated the expression of miR-155, with a consequent increase in inflammatory cytokines, mediated via protein C kinase activated receptor (RACK) 1 [38]. In addition, in a study of LPS-stimulated microglial cells from mice, the flavonoid apigenin inhibited microglial activation and their proinflammatory profile, as well as suppressed the expression of miR-155 in a dose-dependent manner [39].

In this work, we also demonstrated that agathisflavone downregulated the expression of phosphorylated STAT-3 in microglia subjected to inflammatory damage. It is known that the JAK/STAT signaling pathway mediates a variety of biological processes, and its activation has been closely related to inflammatory and autoimmune diseases [40]. Inhibition of JAK2/STAT3 signaling downregulates the expression of inflammatory cytokines, such as IL-1β and IL-6, involving miRNAs such as miR-29b and miR-155, and upregulation of miR-29b has been shown to inhibit IL-1β production through STAT-3 suppression, consequently inhibiting microglia-induced neuronal apoptosis [8,9,10,41,42]. In addition, miR-155 inhibition suppressed cytokine signaling by reducing p-STAT-3 after 7 days of ischemic injury in the cerebral cortex of C57BL/6 mice, reversing this suppression after 14 days of injury [10]. In recent studies, it was shown that LPS stimulation or damage caused in ischemia/reperfusion models in STAT3^f/f^ and STAT3^f/f^ LysM^cre+^ mice could induce phenotypic polarization of microglia through activation of the JAK2/STAT3 pathway [43,44].

In AD, it has been shown that microglia recognize soluble Aβ oligomers through different cell surface receptors, including CD36 and TLRs, such as TLR2, TLR4, TLR6, and TLR9. The recognition by these receptors contributes to the activation of NF-κB, and consequent microglial activation and release of proinflammatory mediators, such as NO, IL-6, and IL-1β [3,4]. Also shown in studies with models of neuroinflammation using LPS, IL-1β, or Aβ oligomers is the effect of agathisflavone and other flavonoids in negatively regulating the expression of NF-κB and, consequently, of the inflammatory cytokines IL-6, IL-1β, TNF, and NOS2, contributing to the reduction of neuroinflammation [3,24,25,26]. Moreover, we also demonstrated that agathisflavone protected neurons against the cytodestructive and proinflammatory effects of IL-1β, a key cytokine that is released by activated microglia via downregulation of NF-κB expression, an effect not observed in combined treatment with α and β estrogen receptor (ER) antagonists [24]. Additionally, other studies carried out in our group have already shown evidence that agathisflavone can interact with the ER, suggesting that this interaction is associated with the inhibition of microgliosis and the release of inflammatory cytokines [22,23]. We also know that STAT3 is a transcription factor that is activated by estrogen, and that agonists for ER receptors promote anti-inflammatory effects [41,42]. In this sense, we believe this to be a possible route of action for agathisflavone in regulating microglial activation and regulation of inflammatory factors and miRNAs expression.

## 4. Materials and Methods

### 4.1. Microglial Cells Cultures

In this work, we used microglial cells obtained from the cortex of newborn Wistar rats (0–2 days old), and human microglial cells of the C-20 lineage. Microglia of the C-20 lineage was kindly provided by Dr. Henning Ulrich, from the Department of Biochemistry, Institute of Chemistry, at the University of São Paulo (USP). These cells were originally designed and characterized by Garcia-Mesa et al. (2017), and in our work, they were cultivated in DMEM F12 medium as described by the authors [45,46].

Newborn Wistar rats, used to obtain primary cultures of microglia, were provided by the vivarium of the Department of Physiology of the Institute of Health Sciences at the Federal University of Bahia (Salvador, BA, Brazil). All experiments were carried out in accordance with the Ethics Committee on Animal Experimentation of the local Institute of Health Sciences (CEUA protocol No 6731220818). Isolation of microglia was performed according to the protocol established at the Guaza Laboratory at the Instituto Cajal in Madrid as described by Mecha et al. [46]. The brains of newborn Wistar rats were removed aseptically; meninges and blood vessels were removed from each cortex. Then, the material was mechanically dissociated and filtered into a sterile 75 mm diameter Nitex membrane (R&D^®^). The filtered was resuspended in DMEM medium (Island Biological Company—Gibco^®^, New York, NY, USA), supplemented with 10% fetal bovine serum (FBS), 10% serum equine (HS), 4 mM L-glutamine, and antibiotics (100 U/mL penicillin and 100 µg/mL streptomycin, Island Biological Company—Gibco^®^, New York, NY, USA). The cells were cultured on poly-D-lysine (25 µg/mL)-coated flasks (TPP, Zellkultur, Switzerland) in a humidified atmosphere with 5% CO_2_ at 37 °C. Upon reaching confluence (7–10 days), adherent microglial cells were harvested by shaking at 165 rpm at 37 °C for 3 h. Isolated microglia were seeded into 96-, 24-, or 6-well plates at a density of 3 × 10^4^/cm^2^. The experiments were performed 24 h after plating. In all cases, the cells were cultured at 37 °C in 5% CO_2_.

### 4.2. Treatments

Cell cultures were induced to inflammatory damage with β-amyloid oligomers (Aβ, American peptide) at a concentration of 500 nM for 4 h or lipopolysaccharide from *Escherichia coli* (LPS) at a concentration of 1 µg/mL (LPS, Sigma Aldrich, St. Louis, MO, USA) for 24 h. Then, they were treated with the biflavonoid agathisflavone (FAB) extracted from *Cenostigma pyramidale* (Tul.) E. Gagnon & G. P. Lewis (syn: *Poincianella pyramidalis*, *Caesalpinia pyramidalis)*, as previously described [24,47], for another 24 h. The flavonoid was dissolved in dimethyl sulfoxide (DMSO, Sigma Aldrich, St. Louis, MO, USA) at a stock concentration of 100 mM and stored at 4 °C, protected from light.

Cells were exposed to agathisflavone at a final concentration of 1 µM. The final dilution was obtained at the time of treatment, diluting the stock solution directly in fresh culture medium without FBS. The concentration and exposure time adopted followed established protocols and considering our previous studies on the effects of agathisflavone in vitro [23,24,25]. Control cultures were treated with DMSO diluted in culture medium in a volume equivalent to the concentration of agathisflavone (0.001%). After treatments, experimental analyses were carried out with the cell and with the conditioned culture medium (MCM) of the control condition and the other treatment conditions. The preparation and solubilization of β-amyloid peptide (Aβ) from synthetic Aβ1-42 peptide (Sigma Aldrich, St. Louis, MO, USA) was performed in the Neuroenergetic and Neuroprotection Group of the Federal University of Rio Grande do Sul (UFRGS) according to the already established protocol [48,49]. The Aβ aliquots used in this work were provided by the Dr. Eduardo Zimmer group at the Institute of Basic Health Sciences, Federal University of Rio Grande do Sul. Aβ oligomer preparations were made from these aliquots. Thus, aliquots of Aβ were resuspended in 2% dimethylsulfoxide (DMSO; Sigma, St. Louis, MO, USA) to obtain a 5 mM solution. This solution was diluted in sterile PBS to 100 μM and incubated at 4 °C for 24 h. After incubation, the preparation was centrifuged at 14,000× *g* for 10 min at 4 °C to remove insoluble Aβ aggregates (fibrils). The centrifugation supernatant, containing the oligomers, was kept at 4 °C until use. To determine the concentration of oligomers in the preparations, the BCA Kit (Bio-Rad, Hercules, CA, USA) was used by the method of Lowry et al. (1951) [50].

### 4.3. Cytotoxicity Analysis

The MTT viability assay was used to assess cytotoxicity in microglia. For this, cells treated for 24 h with agathisflavone (1 µM) and/or Aβ (500 nM) and/or LPS (1 µg/mL) or in the control condition (DMSO) were incubated with 3-(4,5-dimethylthiazole-2-yl bromide)-2,5-diphenyltetrazolium (MTT; Sigma Aldrich, St. Louis, MO, USA). The cells were cultured in 96-well plates (Kasvi, PR, Brazil) for 24 h. After this period, the cultures were incubated with the MTT solution at a final concentration of 1 mg/mL for 2 h, and then incubated with the solution of sodium dodecyl sulfate (SDS) at 20% (*w*/*v*) and dimethyl formamide (DMF) at 50% (*v*/*v*), at pH 4.7 to promote cell lysis. Then, the plates were kept at 37 °C overnight to dissolve formazan crystals. Cell viability was quantified by converting the yellow MTT into formazan to purple, promoted by mitochondrial dehydrogenases from living cells [51]. For this, the optical density of each sample was measured at 540 nm using a microplate reader. Three independent experiments were carried out for each analysis. The results were expressed as the percentage of viability of the treated groups in relation to the control, which was considered to be 100%.

### 4.4. RT-qPCR for miRNA 

The evaluation of the regulation of miRNA expression was performed using RT-qPCR. The total miRNA (containing soluble miRNAs) was purified from C-20 cells treated for 24 h with agathisflavone (1 µM) and/or β-amyloid (500 nM) and/or LPS (1 µg/mL) or in the control condition (DMSO) as already described by Silber et al., 2008 [52] using the miRNeasy kit (Qiagen, Hilden, Germany) for the cell pellet and the miRNeasy Serum/Plasma Advanced kit (Qiagen, Hilden, Germany) for the cell culture supernatant. Then a quantification was performed by spectrometry through the Nanodrop™ 1000 (Thermo Fisher Scientific, Waltham, MA, USA). For cDNA synthesis, 10 ng of sample was used, and the protocol was followed according to the manufacturer’s recommendations. The miRNAs investigated were miR146a (hsa-miR-146a-5p) and miR155 (hsa-miR-155), which have already demonstrated a relevant effect on the modulation of inflammatory profile of microglia. Amplification was performed in the ABI7500 FAST thermocycler (Applied Biosystem, Foster City, CA, USA) and the SyBR™ Green PCR (Thermo Fisher Scientific, USA) Mastermix was used. The endogenous control RNU1A1 was used to normalize the results. The expression of mRNA levels was calculated using the 2^−ΔΔCT^ method by Schmittgen and Livak, 2008 [53] and analyzed using Graphpad Prism v9 (California, EUA, 2020). The results of at least three independent experiments were considered.

### 4.5. RT-qPCR for Cytokines

To evaluate gene expression for proteins of interest, after the treatment period, the culture medium was removed and then total RNA was extracted with Trizol^®^ reagent (Invitrogen, Life Technologies, Carlsbad, CA, USA). Extraction was performed according to the manufacturer’s specifications. Total RNA purity and concentration were determined by spectrophotometric analysis using KASVI Nano Spectrum (cat# K23-0002, PR, Brazil). DNA contaminants were removed by treating the RNA samples with DNase using the Ambion DNA-free kit (cat# AM1906, Life Technologies™, USA). For cDNA synthesis, SuperScript^®^ VILO™MasterMix (cat# MAN0004286, Invitrogen™, Life Technologies, USA) was used in a 20 µL reaction with a concentration of 2.5 µg of total RNA, following the manufacturer’s instructions. Quantitative real-time PCR (qPCR) was performed using Taqman^®^ Gene Expression Assays (Applied Biosystems, CA, USA) containing two primers to amplify the sequence of interest, a specific Taqman^®^ MGB probe and TaqMan Universal Master Mix II with UNG (cat# 4440038, Invitrogen, Life Technologies™, USA). The assays corresponding to the genes quantified in this study were IL1β (Hs01555410_m1), IL-6 (Hs00174131_m1), IL-10 (Hs00961622_m1), and NOS2 (Hs00580555_ m1). Real-time PCR was performed using the Quant Studio 7 Flex™Real Time PCR System (Applied Biosystems, CA, USA). The thermocycling conditions were performed according to the manufacturer’s specifications. The actin beta (ACTB) (Hs00667869_m1) and hypoxanthine phosphoribosyltransferase 1 (HPRT1) (Hs01527840_m1) targets were used as reference genes (endogenous controls) for normalization of gene expression data. Data were analyzed using the 2^−ΔΔCt^ method [53]. The results represent the average of three independent experiments.

### 4.6. Western Blot for STAT3 Proteins

To evaluate the expression of STAT3 signaling pathway proteins by Western blot, microglial cells obtained from the cortex of newborn Wistar rats were cultured in 6-well plates at a density of 1.0 × 10^6^ cells/well. Cultures were induced to inflammatory damage with β-amyloid at a concentration of 500 nM for 4 h and then treated with the biflavonoid agathisflavone (FAB) for 24 h. After this period, total proteins were extracted with 2% (*w*/*v*) SDS extraction buffer, 2 mM ethylene glycol-bis[β-aminoethyl ether]-N, N, N′N′-tetraacetic acid (EGTA), 4 M urea, 0.5% (*v*/*v*) Triton X-100, and 62.5 mM Tris-HCl buffer (pH 6.8), supplemented with 1 μL/ml of a protease inhibitor cocktail (Sigma, St. Louis, MO, USA). Total protein concentration was determined by the method of Lowry et al. (1951) [50], using a protein assay reagent kit (Bio-Rad, Hercules, CA, USA). The proteins were subjected to polyacrylamide gel electrophoresis under denaturing conditions (SDS-PAGE) and transferred to nitrocellulose membranes. Approximately twenty-five micrograms (25 µg) of total proteins were electrophoretically run on 4% stacking gel and 8% running gel (SDS-PAGE). Electrophoresis was performed at 200 V for 45 min. After running, the proteins were then transferred to a nitrocellulose membrane (Bio-Rad, Hercules, CA, USA) at 100 V for approximately 1 h. The presence of proteins in the membrane was confirmed by staining the membrane with Ponceau Red (Sigma). Subsequently, the membranes were blocked for 1h at room temperature in 20 mM Tris-HCl (pH 7.5) containing 0.05% Tween 20 (TBS-T) and 5% skim milk. Subsequently, the membranes were incubated overnight, at room temperature, with the primary antibodies anti-STAT3 (1:1000 Santa Cruz, TX, USA) and anti-pSTAT3 (1:1000 Santa Cruz, TX, USA), anti-α-Tubulina (1:1000, Santa Cruz, TX, USA), under slow agitation, both diluted in TBS-T with 1% milk. The membranes were then washed three times with TBS-T and incubated for 1 h at room temperature with a peroxidase-conjugated anti-rabbit secondary antibody (1:5000; Molecular Probes, OR, USA) diluted in 5% nonfat milk TBS-T. After three washes with TBS-T and one wash with TBS, the membranes were incubated with the chemiluminescent substrate solution (ECL Plus Bio-Rad substrate kit, Hercules, CA, USA) for 5 min. Immunoreactive bands were visualized using the luminescence kit (Bio-Rad, Hercules, CA, USA) according to the manufacturer’s instructions. The quantitative determination of reactive bands was carried out through densitometric analysis of the digitized image, obtained through the image acquisition system (ScanJet 4C—HP), and using the ImageJ 1.4.6u software (Wayne Rasband, National Institute of Health, USA).

### 4.7. Statistical Analyses

The results were analyzed by the GraphPad Prism 9.0 statistical program (Boston, MA, USA) and recorded as median ± standard error of the means (SEM) of the evaluated parameters. To determine the statistical difference between the groups, one-way analysis of variance was performed using the ANOVA test, followed by the Student–Newmann–Keuls post-test for parametric data. For nonparametric data, an analysis was performed using Kruskal–Walis and Dunns post-test. Confidence intervals were defined at a 95% confidence level (*p* < 0.05 was considered statistically significant). In all figures, error bars represent SEM of at least three independent experiments.

## 5. Conclusions

This study provides evidence that miR-146a and miR-155 are important targets of the flavonoid agathisflavone involved in the regulation of microglial activation and neuroinflammation. The results also reinforce the importance of STAT3 signaling in inducing microglial activation in response to neuroinflammatory stimuli. These therapeutic actions of agathisflavone on microglia are relevant to regulating neuroinflammation in multiple neurodegenerative diseases.

## Data Availability

Data are contained within the article.
